# Community Health Workers as Vaccinators: A Rapid Review of the Global Landscape, 2000–2021

**DOI:** 10.9745/GHSP-D-22-00307

**Published:** 2023-02-28

**Authors:** Emily Gibson, Mariam Zameer, Rebecca Alban, Luc Mahougbé Kouwanou

**Affiliations:** a VillageReach, Seattle, WA, USA.; b Centre de Recherche en Reproduction Humaine et en Démographie, Cotonou, Benin.

## Abstract

**Introduction:**

Community health workers (CHWs) could expand immunization access in under-reached communities by administering vaccines. This rapid review identifies countries where CHWs administered vaccines and synthesizes health systems factors that may contribute to or detract from the feasibility of CHWs administering vaccines.

**Methods:**

We conducted a rapid review of peer-reviewed literature from 3 databases and gray literature identified through web searches and by CHW subject matter experts. We treated extracted data on conditions related to vaccine administration by CHWs as qualitative data and conducted deductive content analysis.

**Results:**

We retained 32 documents from 497 initial records and identified 23 CHW cadres that vaccinated in 20 countries, ranging from long-established national programs delivering routine immunizations to pilot projects delivering 1 specific vaccine. CHWs who vaccinate face the following challenges: (1) inadequate supply chain training, (2) inadequate cold chain equipment, (3) transportation for supplies and to communities, (4) heavy existing workload, (5) inadequate or irregular remuneration, (6) inadequate or irregular supervision.

**Conclusion:**

To improve immunization coverage in underimmunized and zero-dose communities, countries where CHWs vaccinate should provide CHWs with adequate remuneration, supervision, supply chain support and management, and formal integration within the health system. CHWs administered vaccines in 20 of the 75 countries with documented CHW programs, suggesting the majority of an estimated 3.3 million CHWs globally do not yet administer vaccines. In light of health care workforce shortages and immunization equity gaps, further exacerbated by the COVID-19 pandemic, policymakers should consider task-shifting vaccine administration to CHWs to bolster immunization access for under-reached communities. Additional systematic documentation is needed to further explore best practices to support CHWs as vaccinators, especially related to supply chain, policy, safety, and efficacy.

## Abstract (precis)

This review identifies countries where community health workers administered vaccines and synthesizes health systems factors that may contribute to or detract from the feasibility of community health workers administering vaccines to alleviate health workforce shortages.

## Teaser

**Key Findings:**

- Community health worker (CHW) cadres administered vaccines in 20 of the 75 countries with documented CHW programs, improving access to immunization services for under-reached communities.
- The review identified several countries where CHWs with brief clinical training and experience were taught to vaccinate, suggesting the feasibility of task-shifting administering vaccines to CHWs with limited experience.

**Key Implications:**

- Through advocacy and funding, international stakeholders should promote equitable treatment of CHWs who vaccinate by ensuring they receive adequate and reliable remuneration, dedicated supervision, and access to transportation.
- Policymakers should advocate for CHWs to be integrated into national health systems as formal health workers, improve data collection for better health system planning, and strengthen supply chain planning and management.

## INTRODUCTION

The Global Vaccine Action Plan aimed to achieve 90% national routine immunization coverage and 80% in every district by 2020.^1^ While global routine vaccination coverage increased from less than 5% to 86% over the past 4 decades,^2^ it has stagnated over the last 10 years.^3^ Between 2010 and 2017, the mortality rate of children aged younger than 5 years decreased by 24%, mainly due to immunization.^3^ Still, many individuals have insufficient access to routine vaccines, including an estimated 25 million infants each year.^4^ The COVID-19 pandemic has further heightened inequities by stalling routine immunization services^5^ as a result of transport disruption, redeployment of the health workforce, and increased risk of COVID-19 at health centers. Routine vaccination coverage decreased from 86% in 2019 to 81% in 2021,^4^ and the number of children not receiving vaccinations increased to 22.7 million, with 17.1 million not receiving a single dose of diphtheria, tetanus, and pertussis (known as zero-dose children),^6^ resulting in increased risk of disease outbreaks.

Low- and middle-income countries face severe challenges to immunization coverage, with a persistently large gap in vaccine access compared to high-income countries.^5^ Access to and availability of vaccines, along with vaccine hesitancy, are the major challenges to increasing vaccination coverage.^7^^,^^8^ Vaccination services rely on health workers to administer them, but a severe health worker shortage in low- and middle-income countries hampers delivery of routine health services, including immunizations.^9^ To address this shortage, the World Health Organization estimates 18 million more health workers are needed by 2030, primarily in low- and middle-income countries.^9^

### A Role for Community Health Workers as Vaccinators

The Immunization Agenda 2030 identifies universal access to immunization services as an essential component of primary health care^10^ and aims to reduce the number of zero-dose children by 50% by 2030. The focus of Gavi, the Vaccine Alliance’s 5.0 strategy is to reach zero-dose children,^11^^,^^12^ who live in consistently missed geographic contexts, such as remote rural areas, urban poor communities, and conflict settings.^13^ They have limited interactions with the public health system and lack the means to access vaccination services.^11^ Community health workers (CHWs), who often live in and are trusted by under-reached communities, could increase equity in vaccine access for underimmunized and zero-dose children with limited access to health care.

Community health workers could increase equity in vaccine access for underimmunized and zero-dose children with limited access to health care.

Many countries rely on CHWs to improve access to primary health care, especially for hard-to-reach populations.^14^ CHWs are a cadre of lay health workers who may be paid or volunteer and work in both rural and urban environments.^15^ The role of CHWs differs notably between countries, but generally, they are considered lay health workers who receive some job training that ranges from several weeks to several years and provide culturally and linguistically appropriate health services to specific communities.^16^ Some countries have several CHW cadres that may have different scopes of practice or may focus on different sub-populations. Contrary to World Health Organization recommendations,^17^ CHWs are often considered an informal health cadre and may work with limited preservice training, supervision, competency-based certification, or pay, with some receiving compensation for time or travel in place of a formal salary.^16^

Decades of research indicate CHWs are trusted agents who help to reduce health disparities by bridging gaps in health care access for underserved communities.^15^ To promote vaccination, CHWs provide education to families, organize vaccination events, remind parents about childhood immunization schedules, and track immunization rates in their communities.^15^ CHWs are effective in improving vaccination coverage; however, this is usually through health promotion and health education activities,^18^^–^^20^ rather than by administering vaccines themselves.

In some countries, like Pakistan and Malawi, CHWs also administer vaccines that have been critical to reaching underimmunized and zero-dose communities. CHWs in Pakistan administer the oral polio vaccine (OPV) as well as bacille Calmette-Guerin, measles, and COVID-19 vaccines in some provinces,^21^^,^^22^ while in Malawi, they administer all routine immunizations.^23^ There are precedents for CHWs administering injections; in at least 20 countries, CHWs administer injectable medication such as contraceptives.^24^^–^^31^

Although much is known about the role of CHWs in improving health system performance, more research is needed to explore opportunities for CHWs to strengthen links between communities and health systems.^32^ CHWs can play an important role in improving vaccine access by administering vaccines; yet before this review, the literature lacked a comprehensive landscape of countries where CHWs are vaccinating and their circumstances. More systematic documentation is needed to inform policy efforts and deploy CHWs as vaccinators to improve vaccine access and equity.

Our rapid review aims to identify conditions and circumstances under which CHWs can provide vaccination, especially injectables. Specifically, the review attempts to describe where and how CHWs are vaccinating and any potential barriers and facilitators. We did not attempt to identify the efficacy or safety of CHWs as vaccinators.

## METHODS

Given the decreasing immunization coverage rates and health workforce shortages, the purpose of this review was to provide timely evidence on the role of CHWs as vaccinators to policymakers and technical partners. Hence, a rapid review was chosen to speed up the systematic review process.^33^ We asked the following research questions: (1) In which countries are CHWs already administering vaccines? (2) What health systems factors contribute to or detract from the feasibility of CHWs administering vaccines?

### Search Strategy and Procedure

The search strategy included peer-reviewed literature, gray literature, and documents supplied by CHW subject matter experts. We included documents published between January 1, 2000 and July 30, 2021 to ensure results addressed recent CHW programs and were applicable to current and future policy. We searched for peer-reviewed literature in PubMed, Cochrane Library (Reviews and Trials), and Web of Science using search terms relating to CHWs, terms relating to vaccination, and terms implying administration of vaccine products. The Box includes a summary of inclusion criteria, and Supplement 1 includes a complete list of search terms, U.S. National Library of Medicine Medical Subject Headings, and Boolean logic as appropriate.

BOX **Inclusion Criteria for Rapid Review of Community Health Workers as Vaccinators**

**Community health workers**: As classified by the “Developing and Strengthening Community Health Worker Programs at Scale” reference guide,^15^ we included documents that referred to community health workers as auxiliary health workers, health extension workers, community health volunteers, lay health workers, or any locally specific term referring to a comparable role.

**Study type**: Qualitative studies, quantitative studies, evaluations, reviews, mixed methods studies, gray literature

**Subject matter**: Community health workers administering vaccines

**Language**: English and French

**Date**: Published between January 1, 2000 and July 30, 2021

We supplemented the search with gray literature from the following websites and organizations: CHW Central, Community Health Impact Coalition, and Gavi databases. We also reached out to experts in the field of CHW research, policy, and programming to solicit documents for review and conducted reference mining of included documents. These subject matter experts were identified using a snowball approach, first through prior publications and by consulting with individuals from well-recognized organizations such as the Community Health Impact Coalition to request additional connections.

### Data Extraction and Analysis

One author screened all titles and abstracts, evaluated documents per the inclusion criteria, and assessed documents for risk of bias using the Joanna Briggs Institute (JBI) critical appraisal tools, a set of tools developed for assessing the quality of published papers.^34^ For those documents included for which there was no applicable appraisal tool, the reviewer adapted an existing tool for the quality assessment. The following data were extracted into a Microsoft Excel spreadsheet: citation; study type; country; CHW cadre; vaccine administration route and type; and health systems factors related to vaccine administration by CHWs under the following 5 predetermined categories: program history, CHW role, supply chain, gender, and occupational conditions (compensation, supervision, and CHW work environment and experiences). Extracted data related to vaccination administration by CHWs were treated as qualitative data, and extracted content was read and re-read by 1 author. We then conducted deductive content analysis; 1 author coded extracted data and identified subcategories within the 5 predetermined categories.^35^^,^^36^ Coded data and sub-categories were reviewed by all authors and refined in an iterative process through discussion. Once categories were finalized, 1 author generated a table organized by country, cadre name, and category to collate extracted data. We then reviewed this table, and all authors participated in an iterative discussion process to identify key findings for each category.

## RESULTS

**FIGURE 1:**
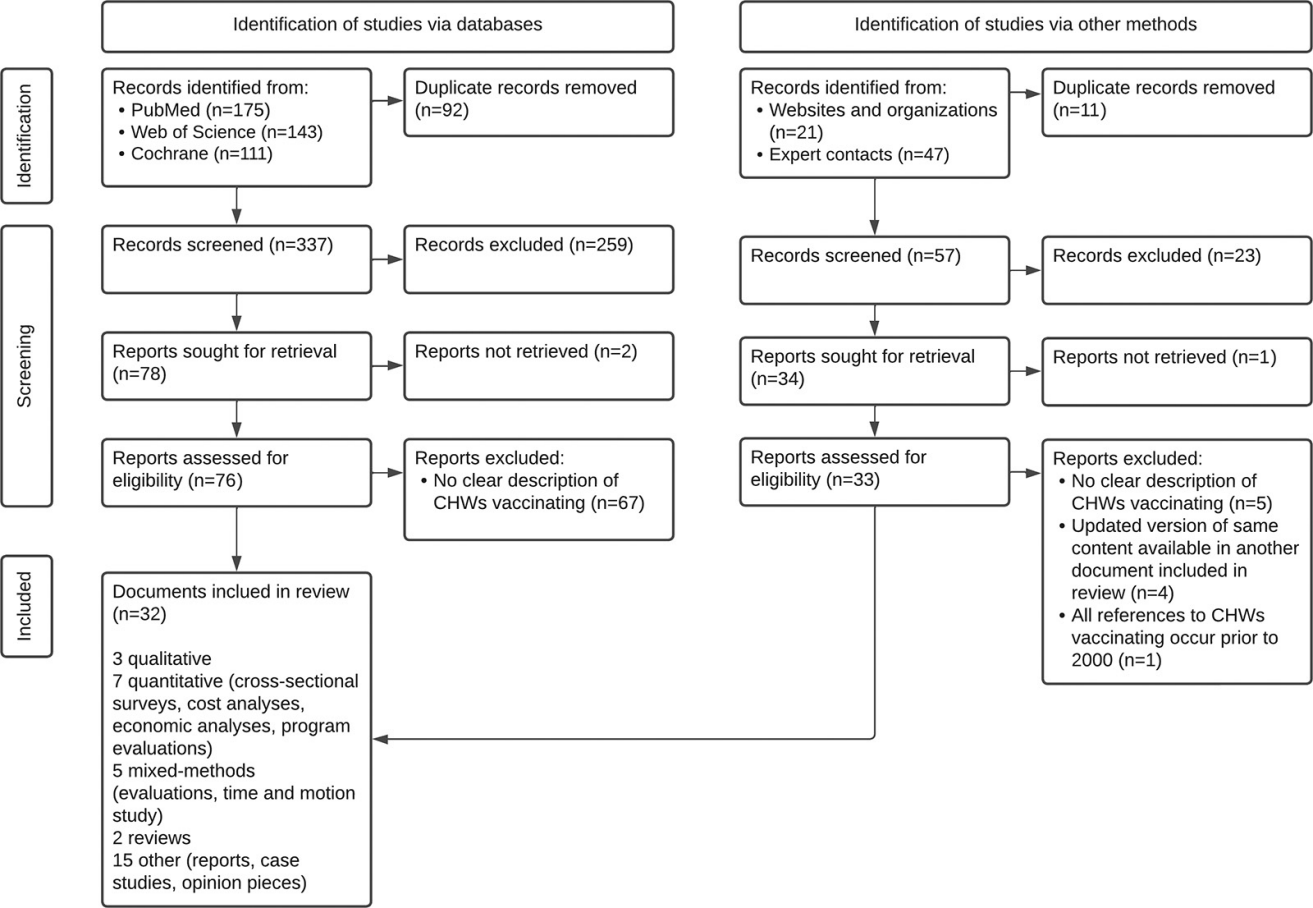
PRISMA Flow Diagram^37^ for a Rapid Review of CHWs as Vaccinators Abbreviations: CHW, community health worker; PRISMA, Preferred Reporting Items for Systematic reviews and Meta-Analyses.

**FIGURE 2:**
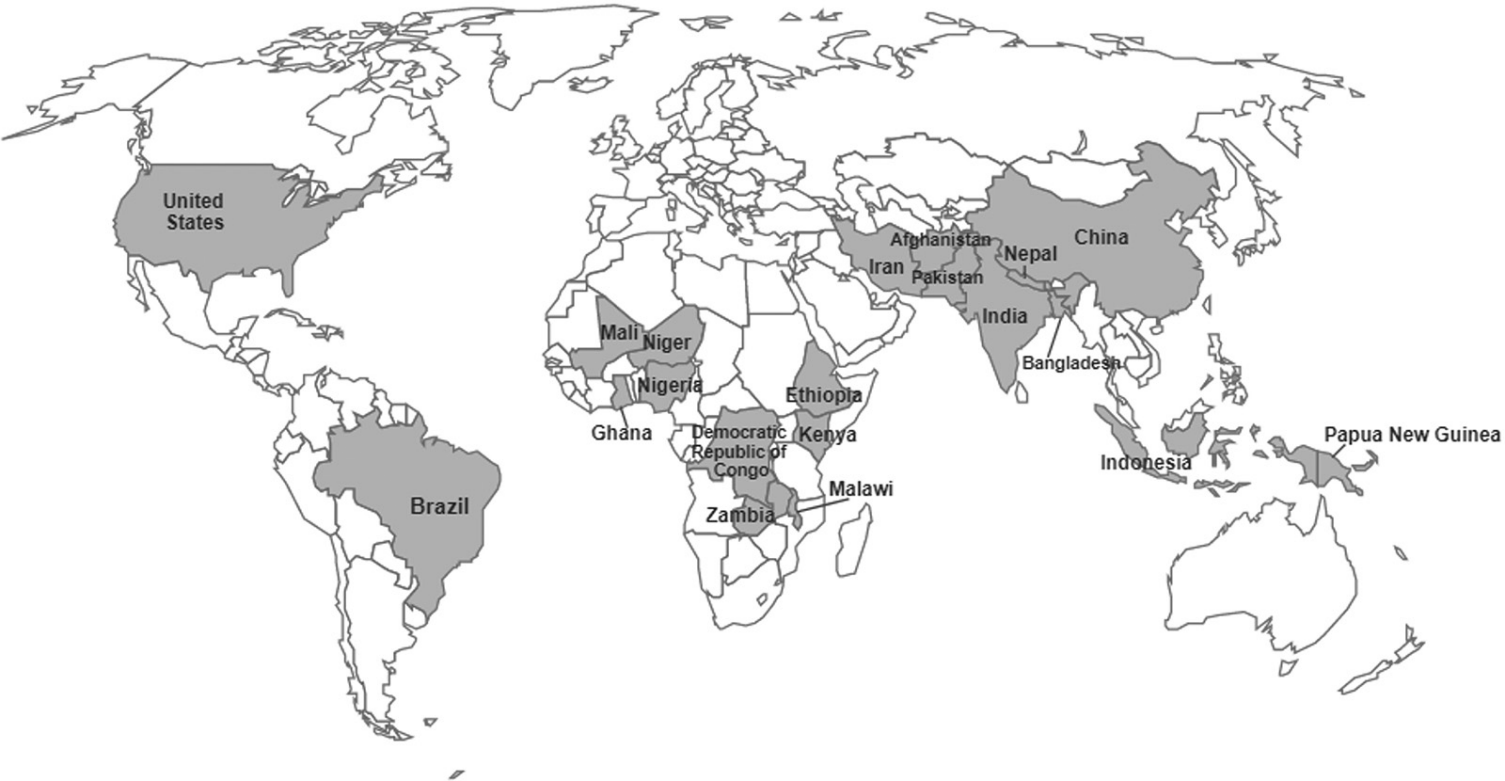
Countries With Community Health Workers Who Administer Vaccines

**FIGURE 3:**
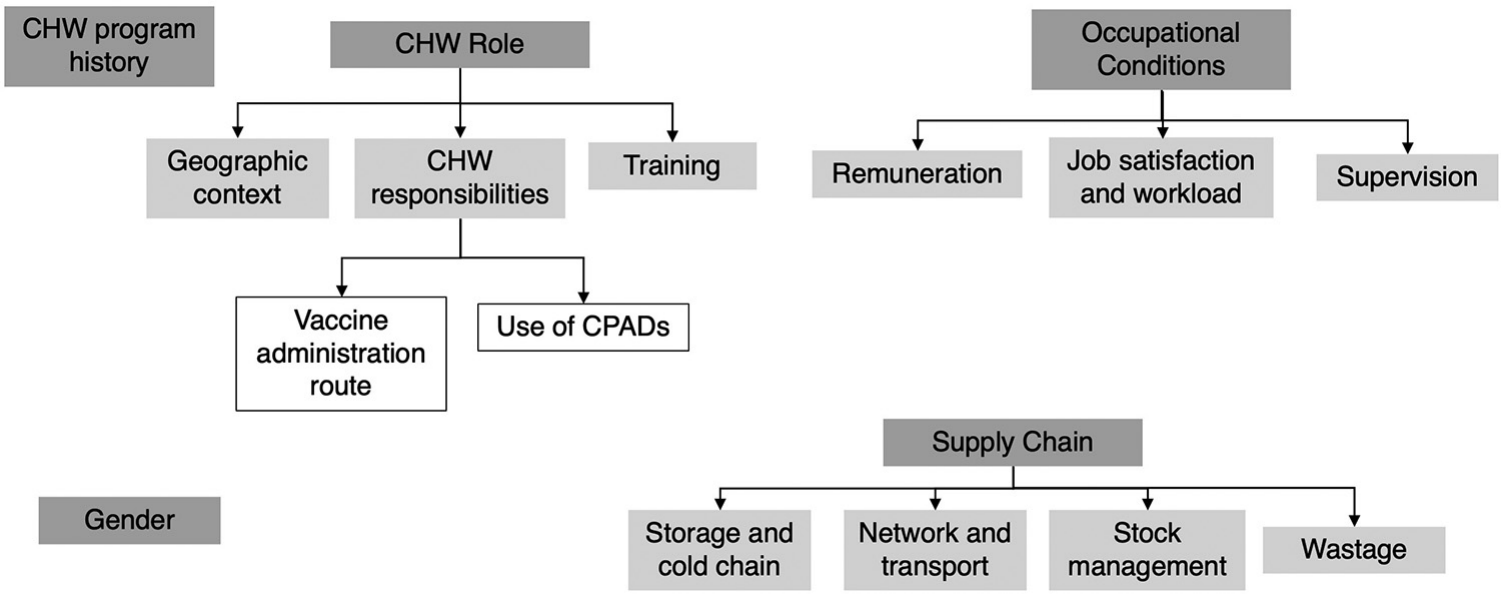
Health Systems Categories Related to CHWs as Vaccinators Abbreviations: CHW, community health worker; CPAD, compact prefilled auto-disable device.

After the screening process and quality evaluation (PRISMA flow diagram outlined in Figure 1^37^), we retained 32 documents for the review from 497 initial records.^21^^,^^23^^,^^38^^–^^67^ The review indicated that 23 CHW cadres administered vaccines in 20 countries from 2000 to 2021 (Figure 2). Within the 5 initial health systems categories used to guide data extraction, content analysis revealed additional subcategories (Figure 3). We list vaccinating CHW cadres and related health systems factors in Table 1 and include the full list of documents in Supplement 2.

**TABLE 1.** CHW Cadres That Administered Vaccines and Related Health Systems Factors, by Country

**Country,** **Vaccinating CHW Cadre, Vaccine Type,** **Program Type**	**Categories**				
	**Program History**	**CHW Role**	**Supply Chain**	**Occupational Conditions**	**Gender**
Afghanistan, TBA, injectable, project or temporary campaign	NR	**Geographic context**: Door-to-door community campaigns.^51^ **Responsibilities**: Tetanus toxoid vaccination campaigns using CPADs.^51^ **Training**: NR	**Storage and cold chain**: NR **Network and transport**: NR **Wastage**: NR **Stock management**: NR	**Remuneration**: NR **Job satisfaction and workload**: NR **Supervision**: NR	NR
Bangladesh, HA, injectable, established program	Initially served as vaccinators or supported malaria control. 1995: expanded to additional preventative health and curative duties. Recruited, trained, and supervised within formal government health system.^59^	**Geographic context**: Rural areas.^59^ **Responsibilities**: Provide women and children immunizations, vitamin A supplementation, and oral rehydration salts; detect and treat TB, diarrhea, malaria, and pneumonia.^47^^,^^59^ Split time between community clinics and home visits.^59^ Other CHW cadres: family welfare assistants, community health care providers, and CHWs employed by NGOs.^59^ **Training**: Requisite 10 years’ schooling; 21 days’ preservice training, on-the-job training.^59^	**Storage and cold chain**: Porters transport the immunization supplies from cold storage centers to the union level.^47^ **Network and transport**: Travel to sites using their own motorcycles or bicycles; given a transport allowance.^47^ **Wastage**: NR **Stock managemen**t: NR	**Remuneration**: Paid full-time workers^47^: government salary US$135–327/month.^59^ **Job satisfaction and workload**: NR **Supervision**: Assistant health inspectors supervise 3 HAs.^59^	Male or female. Historically been male due to the perception that men are more flexible and mobile and the cadre is currently mostly male.^47^^,^^59^
Brazil, community health agent, injectable, established program	Started with promoting immunization, breastfeeding, and oral rehydration solution. Expanded into national public health program. Have an integral role in the system's family health teams to provide primary health care to specific catchment areas.^59^	**Geographic context**: Mainly outside health facility.^59^ **Responsibilities**: Provide preventative and curative services and education (e.g., immunizations, perinatal care, and screening and treatment of infectious and chronic diseases).^59^ Work closely with their family health team (i.e., 4–6 community health agents, nurses, doctors, dentists.)^59^ **Training**: 1,200 hours formal didactic and field activities.^59^	**Storage and cold chain**: NR **Network and transport**: NR **Wastage**: NR **Stock management**: NR	**Remuneration**: Paid, full-time workers: government salary US$226–380/ month.^59^ **Job satisfaction and workload:** Some feel underval-ued by teams, frustrated by low salary and low status, no clear path for career advancement.^59^ **Supervision:** Supervised by nurses on family health team but often missed due to lack of supervising nurses’ time.^59^	NR
China, village-based health worker, injectable, project or temporary campaign	Administration of hepatitis B vaccines was outside usual role of the village-based health workers.^48^^,^^67^	**Geographic context**: Rural areas where many children are born at home.^48^^,^^67^ **Responsibilities**: Administered hepatitis B vaccines using CPADs to infants delivered outside hospital system.^48^^,^^67^ **Training**: NR	**Storage and cold chain**: CPAD stored at room temperature outside cold chain in the homes of village-based health workers or in clinics.^48^^,^^67^ **Network and transport**: NR **Wastage**: NR **Stock management**: NR	**Remuneration**: NR **Job satisfaction and workload**: NR **Supervision**: NR	NR
DRC, volunteer vaccinator, OPV only, established program	Polio vaccine campaign began in 1995 in response to outbreak that involved volunteer vaccinators.^49^	**Geographic context**: Most remote areas of the country.^49^ **Responsibilities**: Deliver OPV and may also deliver other preventative health services and supplies.^49^ **Training**: NR	**Storage and cold chain**: Transport vaccines in vaccine boxes.^49^ **Network and transport**: Must travel long distances carrying vaccine boxes, over challenging terrain using canoes, cars, motorcycles, and bicycles, and on foot.^49^ **Wastage**: NR **Stock management**: NR	**Remuneration**: NR **Job satisfaction and workload**: NR **Supervision**: NR	NR
Ethiopia, HEW, injectable, established program	Formally recognized cadre within government health system with high levels of political support.^59^ Some reports of administrative and managerial challenges.^61^	**Geographic context**: Health posts and on specific days at locations around health posts’ catchment area.^38^^,^^47^^,^^50^^,^^59^^,^^61^ **Responsibilities**: Preventative services (e.g., health education, counseling, FP, and immunization); basic curative services (e.g., first aid and chronic disease management)^38^^,^^40^^,^^41^^,^^47^^,^^50^^,^^59^^,^^61^; administer injectable contraceptives.^40^^,^^50^^,^^61^ Work closely with women's development army (volunteer CHWs that expand HEW reach)^59^ and health center staff.^40^^,^^59^ **Training**: Requisite completion of grade 10, 1 year preservice training^38^^,^^41^^,^^50^^,^^59^; in-service training every 2 years.^59^	**Storage and cold chain**: Federal MOH provides medical equipment, supplies, vaccines and cold chain equipment.^40^ Vaccines transported in vaccine carriers or cold boxes from the health center to outpost. Unused doses brought back at the end of the day.^47^ **Network and transport**: HEWs often travel on foot and sometimes use motorcycles. HEWs are supposed to receive reimbursement for transport but this is sometimes not provided due to shortages in funds.^47^ **Wastage**: NR **Stock management**: Challenges with shortages of drugs, medical supplies, and equipment.^59^^,^^61^	**Remuneration**: Government salary US$84/month.^38^^,^^40^^,^^59^ Can obtain salary increases through additional training and exams.^59^ Per diems and transport reimbursements may not be provided due to lack of funds.^47^ **Job satisfaction and workload**: HEWs may be overburdened due to increasing numbers of tasks assigned.^59^ Lack of a clear career trajectory for HEWs as a challenge.^59^ **Supervision**: Regional health bureaus and district health offices provide support and leadership.^40^ Supervised by health center staff associated with their health post, and sometimes accompanied on vaccination outreach by a health center supervisor.^47^^,^^59^ HEW program evaluations identified lack of regular supervision,^61^ but other reports have noted supervision to be regular.^59^	Women aged over 18 years, usually work at health posts in pairs.^50^^,^^59^
Ghana (1 of 2), CHO/ CHN, injectable, established program	First unpaid CHVs provided immunization and other preventative health tasks. Government transitioned to a paid, more highly trained CHO/CHN cadre.^59^	**Geographic context**: Villages and based out of health posts; also engage in outreach services within their zone.^59^ **Responsibilities**: Preventative services to their CHPS zone (e.g., childhood routine immunizations)^59^ Administered injectable contraceptives.^59^ CHOs supervise CHVs within their zone.^59^ **Training**: All CHOs are first trained as CHNs: 2 years of didactic and field training, with periodic in-service training.^59^ CHNs become CHOs after completing training and orientation in community engagement and mobilization and being posted to a health post.^59^	**Storage and cold chain**: NR **Network and transport**: NR **Wastage**: NR **Stock management**: NR	**Remuneration**: Full-time MOH salary US$140/month. Paid leave and opportunities for paid educational leave.^59^ **Job satisfaction and workload**: NR **Supervision**: Receive facilitative supervision focused on mentoring and problem solving from the GHS from a public health nurse, physician assistant, or designated CHPS coordinator.^59^	NR
Ghana (2 of 2), TBA, injectable, project or temporary campaign	GHS had initial concern about TBAs delivering tetanus vaccines (i.e., this would encourage TBAs to work outside their general scope of practice outside the campaign context, especially as TBAs might benefit financially by offering injection services as they are not officially salaried by the GHS). This was assuaged by ensuring that the training for TBAs to use a CPAD addressed these concerns.^45^	**Geographic context**: Rural area.^45^ **Responsibilities**: Delivered tetanus toxoid injections to women of childbearing age using a CPAD.^45^ **Training**: Before campaign, the TBAs had little or no formal education, although some had prior training in delivering other health care services.^45^ TBAs received training from a few hours to 2 days in delivering the tetanus vaccine using the CPAD.^45^ TBAs reported feeling confident in their ability to safely and correctly use CPADs.^45^	**Storage and cold chain**: NR **Network and transport**: NR **Wastage**: NR **Stock management**: NR	**Remuneration**: Those who participated in the tetanus campaign were unpaid.^45^ **Job satisfaction and workload**: Felt their community status was increased through involvement in the tetanus campaign.^45^ **Supervision**: Supervised during campaigns but some delivered vaccine independently.^45^	NR
India, ANM, injectable, established program	ANMs are official roles within the government health system.^59^ Initially, the ANM role focused on perinatal care and ANMs were expected to stay at the subcenter to conduct deliveries.^52^ The role expanded to be a “multipurpose worker” which included more general maternal and child preventative care along with routine immunizations.^52^ The midwifery component of the role was deprioritized, and ANMs now focus on FP and immunization.^52^^,^^59^	**Geographic context**: Posted at subcenters but many immunization activities are conducted in the community and require the ANMs to travel to villages to conduct outreach sessions with target populations.^52^^,^^59^^,^^60^ **Responsibilities**: Deliver preventative and curative interventions (e.g., routine immunizations, FP, and maternal and child health programming).^59^^,^^60^^,^^62^^,^^64^^,^^66^ ANMs provide care at the subcenter level, conduct home visits, and participate in village health days.^59^^,^^60^^,^^62^^,^^66^ ANMs work closely with other cadres of CHWs, especially AWWs and ASHAs, which both play significant roles in delivery of immunization services, supporting organization of village health days, and community mobilization and education to promote immunization efforts.^59^^,^^62^^,^^64^^,^^66^ **Training**: Must be aged between 17–35 years and have finished 12 years of school to apply to the ANM training program.^59^ 24 months of preservice training.^59^ Some reports found ANMs expressed the need for additional training on a variety of topics including new immunizations.^62^	**Storage and cold chain**: Experience cold chain challenges when conducting vaccinations, including storing vaccines in their domestic refrigerators and limited cold chain staff available for support.^55^ Reports also note a need for additional training in cold chain management and maintenance for ANMs to ensure vaccine potency especially in outreach settings.^55^ **Network and transport**: Report transportation challenges (e.g., limited public transportation and rough terrain).^62^ **Wastage**: NR **Stock management**: ANMs report vaccine shortages as a barrier to administering and promoting vaccines.^64^	**Remuneration**: Salaries vary by state, but are paid a government salary of approximately US$280 per month.^59^ **Job satisfaction and workload**: Considered “multipurpose workers” whose scope requires them to balance time between home visits, health center, and community immunization campaigns.^52^^,^^64^ ANMs are also often required to supervise the health center.^59^ **Supervision**: Lady health visitors supervise ANMs, and there is one lady health visitor for every 6 subcenters.^59^ ANMs can become lady health visitors once they have 5 years of experience and after taking a 6-month training course.^59^ However, reports indicate some ANMs do not feel they receive meaningful supervision.^59^	Must be female to apply to the training program.^59^ Another male cadre of CHWs, multipurpose health workers, sometimes accompany ANMs to remote areas and provide support with transportation. Sometimes ANMs do not feel safe traveling to certain areas without the support of a male multipurpose health worker.^62^ ANMs have been verbally harassed, had stones thrown at them, and been sexually assaulted after being called to village homes, and some refuse to attend house calls at night due to security concerns.^59^
Indonesia (1 of 2), kader, injectable, established program	The community sessions run by the kaders first focused on basic nutrition, growth monitoring, and FP.^59^ The MOH later added additional functions (e.g., to expand immunization coverage and provide curative services).^59^ Reports suggest many kaders have given up their roles out of a need to pursue paid employment, and that many of the community sessions no longer occur.^59^	**Geographic context**: Host sessions at a central location in the community.^59^ **Responsibilities**: Collaboratively run a community health post called a posyandu.^59^ Hold monthly sessions on maternal and child health education and preventative services (e.g., immunization and resupply of FP products).^59^ Supported by local health center staff (i.e., doctors, nurses, and midwives).^59^ **Training**: Less than a week of formal training; many skills acquired through experience.^59^	**Storage and cold chain**: NR **Network and transport**: NR **Wastage**: NR **Stock management**: NR	**Remuneration**: Volunteers, not compensated formally.^59^ May receive reimbursements for travel expenses and informal compensation (e.g., medical treatment).^59^ **Job satisfaction and workload**: Seen to be a highly esteemed community role and may receive awards for years of service.^59^ **Supervision**: Supervised by primary care doctors, nurses, and midwives from the nearby health facility.^59^ At least 1 of those staff members is present for community session run by kaders.^59^	The majority are female, but this is not a requirement.^59^
Indonesia (2 of 2), community midwife, injectable, established program	Trials of community midwives administering the birth dose of hepatitis B were ongoing, but there were issues managing multidose vials and disposable syringes.^51^ 1995: Community midwives piloted use of CPADs to deliver hepatitis B outside the cold chain. 2003: Expanded to target all births in Indonesia.^51^	**Geographic context**: Home visits in villages.^51^ **Responsibilities**: Deliver birth dose of hepatitis B using a CPAD.^51^ **Training**: NR	**Storage and cold chain**: The hepatitis B dose using CPAD able to be stored outside cold chain in midwives’ homes.^51^ Midwives given safety boxes for disposing used CPADs.^51^ **Network and transport**: Already received travel allowances to perform neonatal home visits before adding hepatitis B injection requirement.^51^ **Wastage**: CPAD helped prevent wastage.^51^ **Stock management**: NR	**Remuneration**: Travel reimbursement to conduct home visits.^51^ **Job satisfaction and workload**: NR **Supervision**: NR	NR
Iran, behvarzes, injectable, established program	Government established a lay health cadre to administer immunizations and manage communicable diseases comprised of men with at least 6 years of general education who were provided with a short additional training course.^59^ Government piloted several different models for providing public health care to rural areas, which was eventually developed into the health house model staffed by behvarzes beginning in 1979.^59^^,^^63^	**Geographic context**: Rural areas.^59^^,^^63^ **Responsibilities**: Provide a range of preventative and curative community health services for adults and children (e.g., routine immunization) and engage in community mobilization and engagement.^59^^,^^63^ 1–2 behvarzes run health houses (small rural health outposts).^59^ Behvarzes partner with a second behvarz at health house.^59^^,^^63^ **Training**: Must have completed 12 years of general education followed by 2 years’ didactic and practical training.^59^ More recently, many also have an undergraduate degree.^59^ They are offered monthly refresher courses.^63^	**Storage and cold chain**: NR **Network and transport**: NR **Wastage**: NR **Stock management**: NR	**Remuneration**: Government salary ∼ US$350/month (may increase based on performance).^59^ Additional incentives (e.g., training funds, awards, celebrations, and loans).^63^ Free training; funds for transportation, food, and accommodation during 2 years of training.^59^ **Job satisfaction and workload**: NR **Supervision**: Supervised by higher-level staff from the local comprehensive health centers who regularly visit the health houses.^59^^,^^63^	Both men and women are eligible; health houses often staffed by 1 man and 1 woman from each village.^59^^,^^63^ Priority is given to workers from the community in which the health house is located or to women whose husbands have a permanent position in the village.^59^
Kenya (1 of 2), CHW, injectable, project or temporary campaign	NR	**Geographic context**: NR **Responsibilities**: Provide maternal and child-focused preventative and basic curative services including childhood immunization and family through community-based nonprofit Lwala Community Alliance.^46^ **Training**: NR	**Storage and cold chain**: NR **Network and transport**: NR **Wastage**: NR **Stock management**: NR	**Remuneration**: Salaries paid by Lwala Community Alliance of US$200/month and includes supplies and supervision, although it’s unclear what percentage of that amount goes directly to the CHW.^46^ **Job satisfaction and workload**: NR **Supervision**: NR	NR
Kenya (2 of 2), CHV, OPV only, project or temporary campaign	NR	**Geographic context**: In an effort to reach nomadic families and those living in remote settlements, CHVs administered OPV to children at boreholes and water access points in rural communities.^39^ **Responsibilities**: As part of an oral polio SIA, CHVs were trained to administer OPVs to children in remote areas.^39^ Social mobilization was led by members of the nomadic communities, while CHVs and health workers administered the OPVs.^39^ **Training**: NR	**Storage and cold chain**: NR **Network and transport:** NR **Wastage**: NR **Stock management**: NR	**Remuneration**: NR **Job satisfaction and workload**: NR **Supervision**: NR	NR
Malawi, HSA, injectable, established program	1960s: Temporary lay health cadre created to provide smallpox vaccines. 1973: New cadre created to address cholera and was later expanded to the role of the HSA to focus on health promotion and preventive services.^23^^,^^59^ Although this was not the case for many years, HSAs are an officially recognized cadre with legal support from the MOH and considered to play an important role in public health system.^58^^,^^59^	**Geographic context**: Health centers, outreach clinics, and during national immunization days organized to reach families in remote areas.^53^^,^^58^^,^^59^^,^^65^ **Responsibilities**: Provide community engagement, social mobilization, and preventative public health services for adults and children.^23^^,^^53^^,^^56^^–^^59^^,^^65^ HSAs are one of the main providers of immunization.^53^^,^^56^^,^^58^ Engage in limited basic curative tasks^23^^,^^59^ and administer injectable contraceptives.^23^ Collaborate with teachers to provide education and promote HPV vaccines among young women.^57^ Malawi has several cadres of CHWs: senior HSAs, CHNs, community midwife assistants, and assistant environmental health officers, and CHVs.^23^ Work closely with community leaders, community organizations, and volunteers.^59^ **Training**: Must be aged at least 19 years, have completed 4 years of education, and speak English and the local language.^59^ 12 weeks’ didactic and fieldwork training^56^^,^^59^ and may receive ongoing refresher trainings.^59^ May have limited opportunities for continuing education.^58^	**Storage and cold chain**: NR **Network and transport**: Often have to travel long distances to reach outreach clinics to deliver immunizations, and many have to walk as they do not have bicycles provided by the health system.^53^^,^^58^ Even for HSAs who are provided with bicycles, they may not be fully functional, or may not work on the hilly terrain they have to travel on.^53^^,^^58^ HSAs may be required to pay for bicycle repairs themselves.^58^ **Wastage**: NR **Stock management**: Often work without gloves due to limited supplies.^58^	**Remuneration**: Receive a government salary of approximately US$63 per month, along with per diems for trainings and events, and some nonfi-nancial incentives such as clothing and a bicycle.^59^ There are some concerns about low remuneration.^58^^,^^59^ **Job satisfaction and workload**: Overburdened with tasks due to balancing requirements related to immunization with other community engagement needs.^23^^,^^53^^,^^57^^–^^59^ There is concern that some HSAs have taken on additional curative tasks without adequate training.^23^ HSA vaccination targets are used as performance measures, which can place pressure on HSAs.^58^ When schools closed during the COVID-19 pandemic, HSAs did some social mobilization tasks to promote HPV vaccines.^57^ HSAs have limited opportunities for career advancement and continuing education and limited incentives and remuneration.^58^^,^^59^ **Supervision**: Supervised by senior HSAs monthly and assistant environmental health officers quarterly.^59^ There is some concern that HSAs do not have adequate supervision.^23^^,^^53^	NR
Mali, TBA, injectable, project or temporary campaign	There was initially some pushback from policymakers against TBAs administering tetanus toxoid injections using a CPAD independently as part of routine immunization, so TBAs first administered the vaccine under close supervision by health care personnel in a community campaign setting.^45^	**Geographic contex**t: Door-to-door in communities in rural areas.^45^^,^^51^ **Responsibilities**: Administer tetanus toxoid injections using a CPAD.^45^^,^^51^ **Training**: Limited or no previous education, and many received some training on delivery of health care services.^45^ Received between a few hours to 2 days training on using a CPAD.^45^^,^^51^	**Storage and cold chain**: NR **Network and transport**: NR **Wastage**: NR **Stock management**: NR	**Remuneration**: Not paid but provided with transportation and food during the training on the use of the CPAD.^45^ **Job satisfaction and workload**: NR **Supervision**: Initially closely supervised by health care personnel when administering tetanus toxoid in community campaigns.^45^	NR
Nepal, AHW, injectable, established program	Nepal MOH has had several iterations of CHW cadres to address rural health needs beginning with VHWs who were primarily male and whose main task was to administer immunizations.^47^^,^^59^ VHWs transitioned into AHWs, either replaced as they retired or given additional training to meet AHW requirements.^47^^,^^59^	**Geographic context**: Work from health posts located in villages.^59^ **Responsibilities**: Administer vaccines.^47^^,^^59^ Partner with other health worker cadres (e.g., ANMs and part-time female community health volunteers),^59^ who primarily engage in education and social mobilization (e.g., promoting immunizations).^59^ **Training**: 15–18 months of preservice training.^47^^,^^59^	**Storage and cold chain:** NR **Network and transport**: NR **Wastage**: NR **Stock management**: NR	**Remuneration**: Paid government workers.^59^ **Job satisfaction and workload:** NR **Supervision**: NR	NR
Niger, agent de santé communautaire, injectable, established program	MOH initially created CHW cadres with limited training focused on education and emergency response. 2000: government expanded focus on providing rural health services and then introduced the agent de sante communautaire and relais volunteer roles.^59^	**Geographic context**: Stationed at health posts located in communities at least 5 km from nearest hospital or health center.^59^ **Responsibilities**: Provide preventative services (e.g., immunizations and FP), some basic curative care,^59^ and administer injectable contraceptives.^59^ Work closely with and supervise relais volunteers, volunteer cadre based at same health post but who focused on health promotion and community mobilization for immunization campaigns outside the health posts in villages.^59^ **Training**: Must have completed primary school education, but many have a higher level of education.^59^ 6 months of preservice training.^59^	**Storage and cold chain**: Get supplies from health center associated with their health post.^59^ **Network and transport**: NR Wastage: NR **Stock management**: NR	**Remuneration**: Salary ∼ US$100/month.^59^ **Job satisfaction and workload**: NR **Supervision**: Supposed to be supervised by staff members from health center, but this may not occur regularly.^59^	Generally male.^59^
Nigeria, CHEW or junior CHEW, injectable, established program	CHW cadres began in 1970s with 2 different regional approaches, one focused on VVHWs coordinated by NGOs and the other CHEWs coordinate by the government.^59^ The public health systems have been restructured to establish village health posts that CHEWs work from, and their training evolved to focus around an extensive book of “standing orders” which outlines their scope of work.^59^ The MOH recently required that all VVHWs be reclassified as community health influencers and promotors service (CHIPS) agents, to standardize training and reduce the silos between different programs.^59^	**Geographic context**: Health centers at least 5 km from the closest government health facility and sometimes in community.^47^ **Responsibilities**: CHEWs and junior CHEWs provide preventative services (e.g., immunizations) and some basic curative services.^47^^,^^59^ VVHWs or CHIPS agents focus on health promotion and education.^59^ CHEWs work closely with and supervise the CHIPS agents who are based out of the same facility as them.^59^ **Training**: CHEWs: 3 years of pre-service training; junior CHEWs: 2 years.^47^^,^^59^	**Storage and cold chain**: For health centers with refrigerators, CHEWs pick up vaccines every month, but for those without, CHEWs pick up vaccines on immunization service provision day.^47^ They transport vaccines in vaccine carriers or cold boxes.^47^ **Network and transport:** NR **Wastage**: NR **Stock management:** NR	**Remuneration**: Government employees ∼ US$281/month.^59^ **Job satisfaction and workload**: CHEWs were supposed to split their time between working from the health center and working out in the community, but due to staffing shortages and increasing workload, they are often unable to leave the health centers to work in the community, and some services were offered infrequently.^47^^,^^59^ Due to a lack of clinicians available in rural areas, they may be under pressure to focus on curative needs.^59^ **Supervision**: CHEWs supervised by staff from nearby health facility.^59^	NR
Pakistan, LHW, injectable, OPV only in some provinces, established program	Launched in 1994 and focused on FP and primary health care. Expanded to support large campaigns to address immunization and some management of communicable diseases.^59^ 2001: MOH approved a policy allowing LHWs to administer injections. 2008: approved policy allowing LHWs to participate in campaigns as EPI vaccinators.^21^ However, LHWs may only be allowed to administer vaccines in certain provinces.^44^	**Geographic context**: May work from a local clinic, from their homes, and door-to-door visiting families.^43^^,^^44^^,^^54^^,^^59^ They may travel to different communities as part of large immunization campaigns.^54^ LHW services focus on rural and poor urban areas.^59^ **Responsibilities**: Provide preventative services focused on maternal and child health and participate in vaccination efforts as social mobilizers, educators, and vaccinators.^21^^,^ ^43^^,^^44^^,^^54^^,^^59^ LHWs play key role in delivering polio vaccines through large campaigns.^43^^,^^54^ Their exact scope of work differs between provinces.^44^ Administer injectable contraceptives.^21^^,^^59^ Collaborate with TBAs and midwives and refer patients to health centers for higher levels of care.^59^ May share vaccination sessions with other vaccinators,^21^ although there may be some tension between LHWs and vaccinators.^44^ **Training**: To apply, LHWs must have at least 8 years of education and be aged 18–50 years.^59^ 15 months of didactic and fieldwork training and yearly refresher trainings.^59^ LHWs receive extensive training specific to vaccination, and many feel confident administering intramuscular injections.^21^ There may be some gaps in LHW training related to specific vaccines.^44^	**Storage and cold chain**: Obtain vaccines primarily from health centers and other vaccinators.^21^ They are not provided with vaccine carrier boxes or transportation support and must make their own arrangements for cold chain management.^21^ **Network and transport**: NR **Wastage**: NR **Stock management**: NR	**Remuneration**: Government salary ∼ US$90–180/month,^43^^,^^59^ although there are concerns that remuneration is not adequate and unreliable.^43^ **Job satisfaction and workload**: Some concern the addition of immunization to their standard tasks may overload LHWs.^44^^,^^54^ Some LHWs have advocated for increased benefits and boycotted participating in vaccine activities due to experiences of violence in communities and not being paid for several months.^59^ **Supervision**: LHWs are associated with a public health clinic and are supervised by lady health supervisor from that clinic at least once per month.^59^ LHWs participating in immunization sessions under EPI may also be supervised by area vaccinators and the district health management team.^21^	LHWs are all women,^21^^,^^43^^,^^44^^,^^54^^,^^59^ and preference is given to married applicants.^59^ Government deploys LHWs, as a cadre of women, in vaccination campaigns as male staff will often not be admitted into some parts of the family home.^54^ LHWs sometimes travel away from their communities to participate in campaigns in areas with few LHWs.^54^ If female LHWs are traveling unaccompanied by their husbands, they may be perceived poorly by the community including being targets of verbal and physical abuse and being refused entry into family homes.^54^ LHWs were targeted by militant groups while participating in polio vaccine campaigns.^43^^,^^59^ LHWs are sometimes offered police escorts, but some decline them as they feel it draws attention and makes them more of a target.^43^
Papua New Guinea, village health volunteer, injectable, project or temporary campaign	This program was organized by an NGO in partnership with local communities and local government.^45^ Some village health volunteers were nervous about the additional responsibility related to their new role as vaccinators, so were provided with a copy of a formal letter authorizing them to administer this vaccine from the department of health.^45^	**Geographic context**: Infants’ homes and at local clinics in rural areas.^45^ **Responsibilities**: Administered the birth dose of hepatitis B vaccines using CPADs to infants within the first 24 hours of birth and^45^ delivered other preventative services (e.g., education and distribute medications).^45^ **Training**: 6 weeks of training and^45^ 1-day training on delivering hepatitis B vaccine using a CPAD.^45^	**Storage and cold chain**: NR **Network and transport**: NR **Wastage**: NR **Stock management**: NR	**Remuneration**: Unpaid volunteers but during training given accommodation, food, small stipends, and commodities (e.g., soap and salt).^45^ **Job satisfaction and workload**: Addition of vaccinating task increased motivation to visit infants immediately after birth.^45^ **Supervision**: Supervised by project officer every 1–3 months.^45^	Male and female.^45^
United States, CHA/P, injectable, established program	NR	**Geographic context**: Rural communities in Alaska.^42^ **Responsibilities**: Provide health education and promotion, preventative services (e.g., immunizations), and interventions in emergency settings.^42^ CHA/Ps may be the only health care worker in their community.^42^ **Training**: 3–4 weeks.^42^	**Storage and cold chain**: Have some limitations on their refrigeration capacity at clinics and are unable to store certain vaccines such as the varicella-zoster vaccine due to not meeting the cold chain requirements.^42^ **Network and transport**: NR **Wastage**: NR **Stock management**: NR	**Remuneration**: NR **Job satisfaction and workload**: NR **Supervision**: NR	NR
Zambia, CHA, injectable, established program	CHW cadres were initially not formally regulated, had many different titles, were trained for different lengths of time by different organizations, and had different scopes of work.^59^ Zambia then chose to formalize a paid CHW cadre, partially based on Ethiopia’s HEW cadre.^59^	**Geographic context**: Supposed to split their time between health posts and community.^59^ **Responsibilities**: Provide both preventative and some basic curative services including childhood immunizations.^59^ Work closely with other health posts staff (e.g., nurses, environmental health technicians, community development assistants, and social welfare volunteers who focus on education).^59^ **Training**: Applicants much have completed at least 12 years of education and be aged 18–38 years.^59^ 1 year of preservice training.^59^	**Storage and cold chain**: NR **Network and transport**: CHAs experience transportation challenges.^59^ **Wastage**: NR **Stock management**: Experience unreliable supplies of medication.^59^	**Remuneration**: Government salary ∼US$250/month (includes civil servant benefits).^59^ Given uniform and some equipment (e.g., bicycle).^59^ **Job satisfaction and workload**: Due to staff shortages, CHAs are often required to manage care needs at the health posts and struggle to prioritize community-level work outside the health post.^59^ **Supervision**: Supervised by health staff either at health post or nearest associated health facility.^59^ Supervision is supposed to occur monthly in community but is often deprioritized and rarely happens.^59^	Women are given preference during recruitment process.^59^

Abbreviations: AHW, auxiliary health worker; ANM, auxiliary nurse midwife; ASHA, accredited social health activist; AWW, anganwadi worker; CHA, community health assistant; CHA/P, community health aide/practitioner; CHEW, community health extension worker; CHN, community health nurse; CHO, community health officer; CHPS, Community-Based Health Planning and Services; CHV, community health volunteer; CHW, community health worker; CPAD, compact prefilled auto-disable device; DRC, Democratic Republic of the Congo; EPI, Expanded Programme on Immunization; FP, family planning; GHS, Ghana Health Service; HA, health assistant; HEW, health extension worker; HPV, human papillomavirus; HAS, health surveillance assistant; LHW, lady health worker; MOH, ministry of health; NGO, nongovernmental organization; NR, not reported; OPV, oral polio vaccine; SIA, supplementary immunization activity; TBA, traditional birth attendant; VHW, village health worker; VVHW, volunteer village health worker.

### Program History

Many CHW programs gradually increased the scope of work for CHWs over time to include additional tasks, often connected to increasingly formal training, remuneration, and integration into the government public health system.^21^^,^^23^^,^^47^^,^^52^^,^^58^^,^^59^^,^^61^^,^^63^ In 5 countries, CHWs administered vaccines from the earliest days of the national program (Table 2).^23^^,^^47^^,^^59^ For other countries, there was no information available on when vaccine administration was added to the CHW scope of work. Overall, we found little discussion of the policymaking process that authorized CHWs to administer vaccines; Pakistan is the only country for which a clear policy process was described.^21^ Of the 23 CHW cadres identified in the review, 16 cadres administered vaccines as part of an established government program often at a national scale, and 7 cadres were part of a project or temporary campaign (Table 2).

**TABLE 2.** Community Health Worker Vaccine Administration Responsibilities

**Country**	**Community Health Worker Cadre**	**Program Type**		**Vaccine Administration Route**		**Category of Vaccines Administered**	
		**Established Program**	**Project or Temporary Campaign**	**Injectable**	**Oral**	**All Routine Immunizations**	**1 Specific Immunization** ^a^
Afghanistan	Traditional birth attendant^51^		X	X^b^^,^^c^			X
Bangladesh	Health assistant^47^^,^^59^	X		X		X	
Brazil	Community health agent^59^	X		X		X	
China	Village-based health workers^48^^,^^67^		X	X^c^			X
Democratic Republic of the Congo	Village volunteer^49^	X			X		X
Ethiopia	Health extension worker^38^^,^^40^^,^^41^^,^^47^^,^^50^^,^^59^^,^^61^	X		X		X	
Ghana	Community health officer^59^	X		X		X	
	Traditional birth attendant^45^		X	X^c^			X
India	Auxiliary nurse midwife^52^^,^^55^^,^^59^^,^^60^^,^^62^^,^^64^^,^^66^	X		X		X	
Indonesia	Kader^59^	X		X		X	
	Community midwives^51^	X		X^c^			X
Iran	Behvarzes^59^^,^^63^	X		X		X	
Kenya	Community health worker^46^		X	X		X	
	Community health volunteer^39^		X		X		X
Malawi	Health surveillance assistant^23^^,^^53^^,^^56^^–^^59^^,^^65^	X		X		X	
Mali	Traditional birth attendant^45^^,^^51^		X	X^c^			X
Nepal	Auxiliary health worker^47^^,^^59^	X		X		X	
Niger	Agent de santé communautaire^59^	X		X		X	
Nigeria	Community health extension worker^47^^,^^59^	X		X		X	
Pakistan	Lady health worker^21^^,^^43^^,^^44^^,^^54^^,^^59^	X		X	X	X	
Papua New Guinea	Village health volunteer^45^		X	X^c^			X
United States	Community health aide/practitioner^42^	X		X		X	
Zambia	Community health assistant^59^	X		X		X	

a Trained as vaccinators to administer 1 specific vaccine (e.g., only hepatitis B vaccines).

b An X indicates clear affirmative evidence is available in documents included in review; a blank cell indicates no information was available in included documents.

c Use of a compact prefilled auto-disable device.

Many CHW programs gradually increased the scope of work for CHWs over time to include additional tasks.

### CHW Role

#### Geographic Context

In every country identified in this review, the CHW cadres that administered vaccines worked in nonurban contexts (e.g., villages and rural areas).^38^^,^^39^^,^^42^^,^^45^^,^^47^^,^^48^^,^^50^^–^^53^^,^^58^^–^^60^^,^^63^^,^^67^ They administered routine immunization at health facilities and in the community, which may involve going door-to-door.^38^^,^^43^^,^^47^^,^^50^^,^^52^^–^^54^^,^^58^^–^^61^^,^^63^^,^^65^ CHW cadres that administered 1 specific vaccine conducted primarily door-to-door visits.^45^^,^^48^^,^^51^^,^^67^

#### CHW Responsibilities

The CHW’s role in many countries focuses on health education and community mobilization. However, the responsibilities of CHW cadres that administered vaccines often included providing other health services, such as first aid or detecting and treating certain medical conditions like TB, malaria, and pneumonia.^23^^,^^42^^,^^45^^–^^47^^,^^59^^,^^63^ Fifteen CHW cadres administered all routine immunizations as part of their core responsibilities (Table 2). Eight CHW cadres were trained to administer 1 vaccine as part of a specific vaccination initiative, such as village-based health workers being trained to administer the birth dose of hepatitis B (Table 2).^48^^,^^67^ Five of these 8 cadres only administered vaccines as part of a project or temporary campaign, and from included documents, it was not clear if vaccine administration by CHWs continued after conclusion of the initial project or was established as part of national immunization policy. However, these 8 cadres provide examples of an established CHW cadre’s responsibilities expanding to include vaccine administration, accomplished by introducing 1 specific vaccine to the scope of a CHW cadre that previously did not administer them. In 5 of the 20 countries where CHWs administered vaccines, CHWs also administered injectable contraceptives.^21^^,^^23^^,^^40^^,^^50^^,^^59^^,^^61^

Most routine immunizations are delivered via injection, but some are delivered via an oral route. Of the 23 vaccinating cadres identified in this review, 21 cadres administered injectable vaccines, and 2 cadres administered oral vaccines only (Table 2). In Kenya, different CHW cadres have different scopes related to administration route: unpaid community health volunteers administered OPV to rural communities, while paid CHWs administered routine injectable vaccines.^46^ In the Democratic Republic of the Congo, volunteer vaccinators only administered OPV.^49^ No information was included about whether the CHW cadres that administered injectable vaccines also administered oral vaccines, except for those in Pakistan. Lady health workers (LHWs) in Pakistan administer injectable vaccines in some provinces, along with OPV.^44^ Additionally, no information was included on adverse events following immunization related to CHWs administering vaccines.

Many health systems include several cadres of health workers classified as CHWs, and each may have a slightly different clinical scope. For example, one cadre may conduct health assessments and administer medications, while another may conduct community outreach and health education. Eleven countries included in this review had multiple CHW cadres.^21^^,^^23^^,^^39^^,^^46^^,^^51^^,^^59^^,^^62^^,^^64^^,^^66^ In 8 of these countries, only 1 CHW cadre administered vaccines.^21^^,^^23^^,^^59^^,^^62^^,^^64^^,^^66^ In these cases, the CHW cadre with the highest clinical scope or a role more focused on health care delivery administered vaccines, while other CHW cadres focused on health education, social mobilization, and social support. In India, there is evidence of close collaboration with the other CHW cadres that supported immunization efforts through community mobilization and organizing of vaccination events.^39^^,^^59^^,^^62^^,^^64^^,^^66^

In countries with several cadres of CHWs, the cadre with the highest clinical scope or a role more focused on health care delivery administered vaccines.

In the other 3 countries—Kenya, Indonesia, and Ghana—with multiple CHW cadres, there were 2 CHW cadres that vaccinated.^39^^,^^45^^,^^46^^,^^51^^,^^59^ In these 3 countries, 1 cadre administered routine immunizations, and the other administered 1 specific vaccine during campaigns: tetanus toxoid vaccines to women of reproductive age in Ghana,^45^ the birth dose of hepatitis B vaccine to newborn infants in Indonesia,^51^ and OPV as part of supplemental immunization activities in Kenya.^39^

No CHW cadre focused exclusively on administering immunizations; however, in Malawi and the Democratic Republic of the Congo, the initial concept of the CHW program consisted of a cadre that only administered vaccines.^23^^,^^59^

#### Use of Compact Prefilled Auto-disable Devices

Administering an injectable vaccine generally requires several steps: (1) acquiring injection materials and maintaining appropriate cold chain requirements for the vaccine vial, (2) reconstituting a vaccine, (3) drawing up the correct dose in a syringe from a multidose vial, (4) administering the injection, and (5) safely disposing of the injection materials.^45^ Compact prefilled auto-disable devices (CPADs) simplify the injection process by eliminating the need to reconstitute the vaccine and draw the correct dose^45^^,^^68^ and make vaccination easier for health workers who are not accustomed to administering vaccines.^45^ CPADs cannot be reused, decreasing the risk of bloodborne infection transmission. They can reduce open vial vaccine wastage by eliminating the need to open a multidose vial when only a single dose is required.^45^^,^^68^ Certain CPAD vaccines, such as the hepatitis B Uniject vaccine, are also occasionally stored outside the cold chain,^48^^,^^51^^,^^67^ especially when used in conjunction with a vaccine vial monitor, although this is considered off-label use.

CHWs used CPADs to administer vaccines in 6 countries: the birth dose of hepatitis B vaccine in China,^48^^,^^67^ Indonesia,^51^ and Papua New Guinea,^45^ and tetanus toxoid vaccines for women of reproductive age in Afghanistan,^51^ Ghana,^45^ and Mali.^45^^,^^51^ All 6 CHW cadres who used CPADs did not previously administer immunizations and used CPADs to administer 1 specific vaccine. In Indonesia, after piloting the use of CPADs with community midwives, the government ultimately implemented the program on a national scale. For the other 5 countries where CHWs used CPADs, it was unclear from the included documents if CHWs continued to administer vaccines beyond the initial research or project timelines. There is also evidence of CHWs administering other injectable medications, such as antibiotics and oxytocin, using CPADs.^45^^,^^68^

### Training

For CHWs administering routine vaccines, their training ranged from less than a week of formal training for kaders in Indonesia to 2–3 years for community health extension workers in Nigeria.^59^ There were no data on the training content or if and to what extent training on vaccine administration was included. The only documents that described training specifically for vaccine administration referred to programs that trained CHWs to use CPADs to administer 1 specific vaccine.^45^^,^^51^ These supplemented existing training and ranged from 2 hours to 2 days.^45^

### Occupational Conditions

#### Remuneration

**FIGURE 4:**
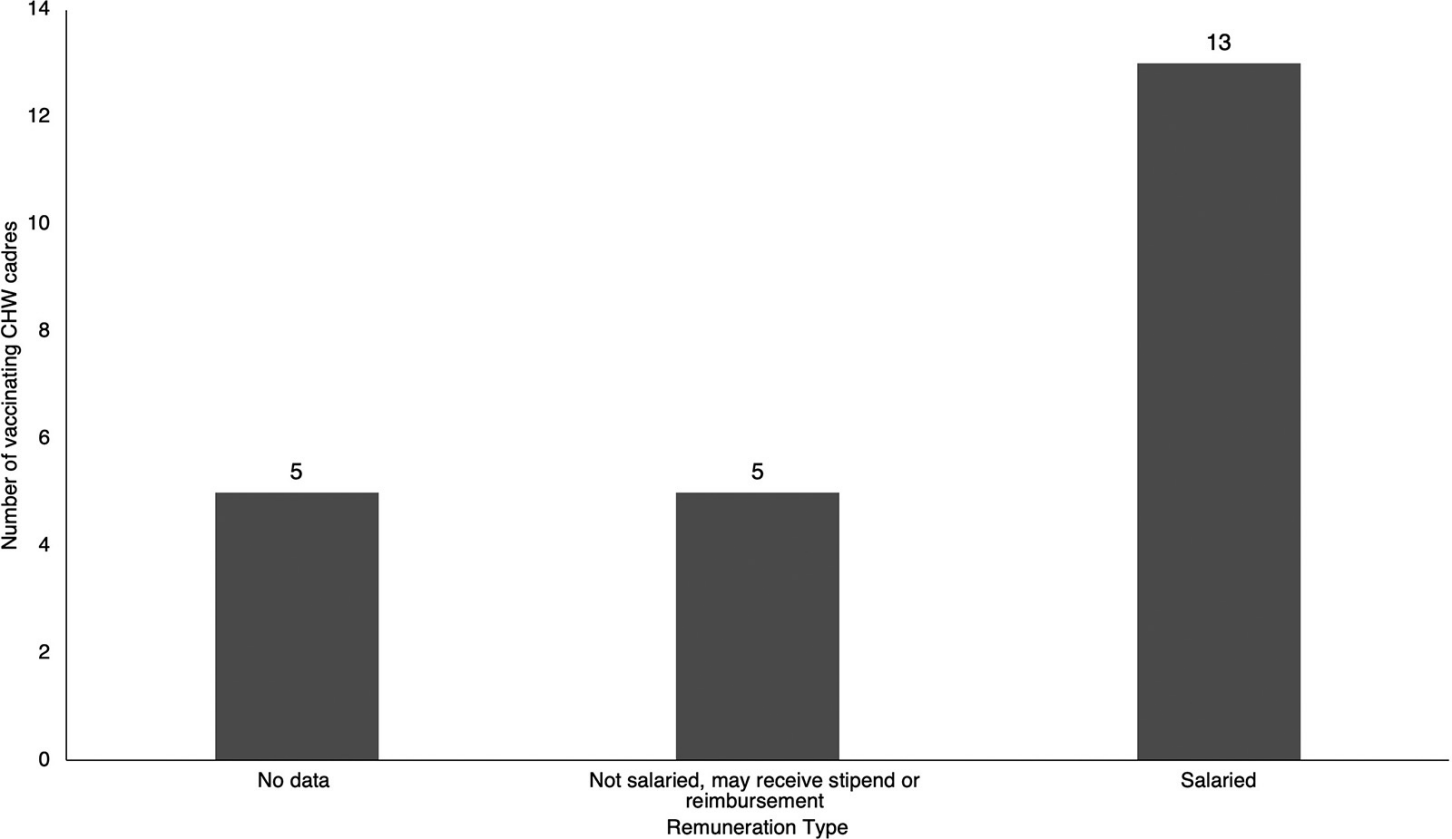
Remuneration for CHWs Who Administer Vaccines Abbreviation: CHW, community health worker.

Remuneration information was available for 18 of the 23 CHW cadres included in the review (Figure 4), of which 13 cadres were salaried employees and received a monthly salary between US$63 per month in Malawi and US$380 per month in Brazil.^59^ Twelve of these salaried cadres were paid by the government, and 1 was paid by an NGO. Some CHWs also received other forms of compensation or benefits in addition to their monthly salary, such as a uniform, medical equipment, bicycles, per diems, transport reimbursements, paid leave, paid education opportunities, and loans.^47^^,^^59^^,^^63^

Several CHW cadres—kaders and community midwives in Indonesia,^51^^,^^59^ traditional birth attendants in Ghana and Mali,^45^ and village health volunteers in Papua New Guinea^45^—did not receive a salary but may have received stipends or reimbursement for travel expenses and medical treatment, free transportation, food, or accommodation during their training period. Many CHWs, whether salaried or reimbursed, encountered issues with remuneration, such as delayed or missing payments.^43^^,^^47^^,^^58^^,^^59^

Many CHWs, whether salaried or reimbursed, encounter issues with remuneration such as delayed or missing payments.

#### Job Satisfaction and Workload

The personal job experiences of CHWs were mentioned for 10 countries included in this review. CHW cadres administering routine immunization described their experience as follows: under-valued, frustrated, overburdened, underpaid, struggled to balance between time at health posts and time in the community, struggled to manage administering immunizations with other responsibilities, lacked a formal career trajectory, and dealt with staffing shortages.^23^^,^^44^^,^^47^^,^^52^^–^^54^^,^^57^^–^^59^^,^^64^

For CHW cadres administering only 1 specific vaccine, some felt their community status was increased through their involvement in a vaccination campaign, and others experienced increased motivation to conduct home visits due to the addition of their vaccination responsibilities.^45^

#### Supervision

For 15 of the 20 countries identified in this review (Table 1), documents described some type of direct supervision of CHW cadres that vaccinated. CHW supervision may have included mentoring, evaluation, and on-the-job observation and training related to CHW responsibilities. Supervision may have been conducted at health posts or in the community and may have been provided by a senior CHW with additional supervisory training; a higher-level health worker, such as a nurse, doctor, midwife, or physician’s assistant from a nearby health facility; or a staff person from the local or regional health offices.^21^^,^^23^^,^^40^^,^^45^^,^^47^^,^^53^^,^^59^^,^^63^ For several countries, documents included in the review identified concerns that CHW supervision may be irregular, unpredictable, or insufficient.^23^^,^^53^^,^^59^^,^^61^

### Gender

Globally, the majority of CHWs are female (estimates indicate 70%).^69^ Specific information about the gender of CHWs was only included for 9 of the 23 CHW cadres included in this review (Table 1). In Ethiopia, India, Indonesia, Pakistan, and Zambia, the CHWs that administered vaccines were mostly women; in some countries, being female was preferred or required for CHW applicants.^21^^,^^43^^,^^44^^,^^50^^,^^54^^,^^59^ In Bangladesh and Niger, the CHWs were mostly male,^47^^,^^59^ and in Iran and Papua New Guinea, CHW roles were more evenly split between men and women.^45^^,^^59^^,^^63^ There was not enough information in the included documents to indicate patterns in the gender breakdown for CHWs who vaccinated compared to those who did not.

Safety concerns for female CHWs were mentioned for 2 cadres: auxiliary nurse midwives (ANMs) in India and LHWs in Pakistan. ANMs and LHWs, who are all women, have experienced verbal, physical, and sexual abuse while conducting their work in remote areas.^43^^,^^54^^,^^59^ In some areas, a male CHW accompanied ANMs when traveling to remote areas in which they did not feel safe traveling alone.^62^ LHWs may also have been accompanied by their husbands or police escorts while traveling outside their communities, as they may have faced danger or been perceived poorly by outside communities as women traveling alone.^43^^,^^54^^,^^59^

### Supply Chain

#### Storage and Cold Chain

CHWs had limited cold chain storage and training.^42^^,^^55^ If they had a refrigerator at their health post, they could store vaccines there; in cases where they did not, they had to transport vaccines in vaccine carriers or cold boxes each time before a session and return any unused vaccine at the end of the day.^21^^,^^47^^,^^59^ Some CHWs were not provided with vaccine carriers and made their own arrangements,^21^ potentially increasing risk to vaccine potency.

In China and Indonesia, CHWs administered hepatitis B vaccines using a CPAD stored outside the cold chain at room temperature,^48^^,^^51^^,^^67^ which made vaccine storage and transport for immunization sessions easier. In some cases, CHWs stored unrefrigerated vaccines in their homes in safety boxes, allowing for rapid access when delivering a birth dose of hepatitis B vaccine.^51^

#### Network and Transport

Personnel and vaccine supply transportation was also challenging, as many CHWs were located far from facilities where vaccines were stored. CHWs often traveled to pick up vaccines from storage facilities (e.g., health centers) and transported vaccines to the health post or to immunization sessions.^21^^,^^47^^,^^59^ To conduct immunization sessions and delivery of other services, many CHWs traveled long distances by foot, bicycle, motorcycle, canoe, and public transportation and may have faced rough, steep terrain.^47^^,^^49^^,^^53^^,^^58^^,^^62^ They may or may not have been given a bicycle, reimbursed for travel costs, or responsible for bicycle repairs.^47^^,^^53^^,^^58^

#### Wastage

Open vial vaccine wastage reflects the portion of a multidose vaccine vial discarded without being administered. Managing open vial wastage could be particularly challenging in rural communities with lower population density where CHWs often work.^45^^,^^51^ The use of CPADs by CHWs could help reduce open vial wastage in remote areas since each device is prefilled with a single dose and prevents the need to use a multidose vial to deliver 1 vaccine.^51^

#### Stock Management

CHWs experienced shortages of equipment and medical supplies, such as vaccines, medications, and gloves.^47^^,^^58^^,^^59^^,^^61^^,^^64^ No information was included about the participation of CHWs in vaccine or supply ordering and inventory processes, use of logistics management information systems, reporting of wastage rates, or supply chain supervision.

## DISCUSSION

### Global Landscape of CHWs Vaccinating

The 23 CHW cadres in 20 countries identified in this review administered vaccines in rural areas where access to immunization services was often limited. As trusted community members trained to both work in a fixed health post and conduct door-to-door visits, CHWs are well positioned to proactively vaccinate underimmunized and zero-dose populations, especially those living in under-reached communities.

This review identified several countries in which CHWs with brief clinical training and experience were taught to vaccinate. These findings suggest it is feasible to task-shift administering vaccines to CHWs with limited experience, provided they receive supplemental training. Many of the vaccinating CHW cadres identified in this review have a broader scope of practice that includes some preventative and curative interventions, as opposed to a role focused on education and community engagement, as is common for many CHW cadres. While task-shifting vaccine administration may be more straightforward for a CHW cadre with a higher clinical scope, this does not appear to be a prerequisite for vaccinating. Use of CPADs, or a similar device, could facilitate expanding the scope of CHWs who do not currently administer vaccines by simplifying vaccine administration and supply chain processes, minimizing opportunities for dosage errors, and reducing wastage related to multidose vials.

Our findings suggest it is feasible to task-shift administering vaccines to CHWs with limited experience provided they receive supplemental training.

Included documents contained limited information on the political process required to add vaccine administration to the scope of work of an existing CHW cadre. Some cadres included in this review administered vaccines as part of a specific project or research experience, but it was unclear if vaccinating remained part of the cadre’s responsibilities or enshrined in national policy after the projects concluded. However, most CHW cadres’ scope of practice changed over time based on ministry of health priorities, and several countries have a CHW cadre to which vaccine administration was added as a responsibility after its initial establishment. This suggests that if ministries of health were to prioritize CHWs administering vaccines, both oral and injectable, then CHWs’ scope could shift to accommodate this if provided adequate training and support. Some governments may be concerned about protection against blame or retaliation in the event of perceived or actual adverse events related to CHWs vaccinating, although this did not come up in the included documents.

### Barriers Faced by CHWs Who Vaccinate

#### Supply Chain

Supply chain barriers faced by CHWs resemble those faced by all health workers delivering services in under-reached communities, especially in rural areas. Supply chains are often designed for cost-efficiency but often do not consider equity to reach the most underserved communities.^70^ The geographically remote nature of CHWs’ work means they are usually located far from vaccine storage and are often responsible for acquiring vaccines and managing vaccine storage either at their health post or in the community until they are administered. Inadequate cold chain support and training could lead to temperature excursions that can reduce vaccine potency. Vaccines licensed for use in a controlled temperature chain—where certain vaccines are labeled for storage and administration outside the standard cold chain for specific periods of time, under specific conditions—could facilitate CHW vaccine administration, especially in remote areas.^71^

Managing open vial vaccine wastage is particularly challenging in a rural context with decreased population density, where there may be scenarios in which only 1 dose from a 20-dose vial is needed that day, and the remainder must be discarded. To improve vaccine equity, a vaccine vial should be opened to vaccinate even 1 child. However, high open vial wastage rates are a major issue for immunization programs in low-income countries, as wastage can substantially impact the financial viability of immunization programs.^68^

Shortages of basic medical supplies and equipment are also a challenge to immunization delivery and CHW safety. Transportation challenges, such as lack of support to reach rural communities and lack of or delayed transportation reimbursement, are also barriers to CHWs administering vaccines.

#### Occupational Conditions

CHWs also face barriers related to their experience at work, such as being overburdened with tasks, health workforce shortages, and balancing the needs of their health post with time spent in the community conducting immunization. A possible facilitator of CHWs vaccinating could be the existence of 2 or more CHW cadres that can support complementary aspects of immunization. In these situations, 1 cadre focuses on vaccine administration while the other concentrates on education, community engagement, and social mobilization. However, the existence of multiple CHW cadres does not appear to be a necessary precursor to CHWs vaccinating as this was not the case in all countries where CHWs vaccinate.

Vaccinating CHWs also face barriers related to supervision and remuneration. The majority of CHW programs included in this review had a supervision process built into the program that provides support in balancing tasks and acquiring adequate training. However, there are concerns that supervision may be irregular, unpredictable, or insufficient.

Most vaccinating CHW cadres identified in this review are paid government workers, and a small number are unpaid or receive only non-salary compensation such as per diem or transportation reimbursement. However, several vaccinating CHW cadres experience delayed or missing payments despite being salaried. A high proportion of the vaccinating CHW cadres are being paid and exhibited other traits to indicate their integration into the health system in terms of training, supervision, role definition, and financial incentives. Only 14% of CHWs in sub-Saharan Africa receive salaries,^72^ and comparatively, the vaccinating CHWs represented in this review showed stronger linkages to the government for sustainable, reliable pay and support. As such, while some volunteer cadres do administer vaccines, enshrining sustainable payment mechanisms, ideally by the government, may contribute to the long-term feasibility of vaccination by CHWs.

Enshrining sustainable payment mechanisms, ideally by the government, may contribute to the long-term feasibility of vaccination by CHWs.

Safety concerns are a possible occupational hazard, particularly for female CHWs, and a potential deterrent to participation in immunization activities. These concerns could serve as a barrier to CHWs traveling to conduct immunization activities, which often requires entering the homes of families in remote areas or leaving their communities to participate in campaigns.

### Recommendations

We recommend the following to ensure equitable treatment of CHWs who vaccinate and increase immunization access and equity in under-reached and rural communities.

- **Ensure adequate, reliable remuneration for CHWs**. Paying CHWs is considered best practice,^17^^,^^73^ and existing research indicates appropriate financial compensation increases CHW job satisfaction and professional well-being.^74^^,^^75^
- **Include CHWs in supply chain planning, provide supply chain training, and ensure access to adequate cold chain equipment.** In supporting immunization initiatives, especially in rural areas, CHWs who vaccinate manage the final stages of vaccine supply chains. Successful supply chain management is crucial to administer potent vaccines and prevent closed vial vaccine wastage due to temperature excursions.
- **Ensure reliable access to a dedicated CHW supervisor.** Supervision is vital to ensure CHWs have access to necessary training and tools to succeed in their roles. Frequent supportive supervision is an evidence-based strategy for bolstering CHW motivation and improving immunization program quality.^76^^,^^77^
- **Integrate CHWs as formal health workers in national health systems.** Integrating CHWs into national health systems creates an enabling environment for them to be recognized and supported as a professional health cadre and receive certification, standardized pay, training, and professional development opportunities.^17^^,^^77^
- **Maintain accurate national records of CHW demographic data**. Lack of demographic data undermines health system planning and impedes CHWs’ provision of care.^76^^,^^78^ Increased availability of demographic data for CHWs could improve governments’ ability to train, pay, and provide other professional support for CHWs to successfully vaccinate.

We also recommend additional research to conduct a deeper landscape of countries in which CHWs administer vaccines. Partnering with country experts for this landscaping process would be crucial as this review identified a lack of current documentation on whether CHWs are vaccinating, and much of the data included in this review did not come from published literature. Finally, to further understand the needs of and provide adequate support for CHWs who vaccinate, additional research into acceptability of CHWs as vaccinators, necessary supply chain training and support, and the safety and efficacy of CHWs administering vaccines is needed.

### Limitations

This review serves as a first step in expanding and synthesizing available research to provide a global landscape of where and how CHWs are administering vaccines. One key limitation was the scarcity of information available on CHWs vaccinating, and for several CHW cadres only 1 source was available. We included 5 documents despite some quality concerns; these articles were retained to gather information from relevant descriptive sections even if the analytical content was not of the highest quality. Additionally, few of the included documents are specifically focused on the role of CHWs administering vaccines. As CHWs are often considered an informal cadre, their work is not always systematically documented, so there may be countries in which CHWs are vaccinating that were not captured in this review.

Furthermore, this review identified a lack of information on the ways CHWs interface with key aspects of the vaccine supply chain. This is concerning given that supply chain is such an important factor in immunization delivery, regardless of who is administering the vaccine. Potent vaccines need to be available when and where they are needed by communities. There was also limited information regarding the political processes and policy guidelines required to legally allow CHWs to vaccinate. Due to limited availability of demographic data in the included documents, there is no indication that gender-related factors are either a barrier or a facilitator to CHWs vaccinating. The lack of demographic data for CHWs is a widespread issue across countries due to poor data management and inaccurate or frequently unavailable data on the number and location of active CHWs.^78^

Finally, clearly defining CHWs is challenging due to the diversity of definitions between countries. This review considered several cadres as CHWs that some may consider better categorized as a more advanced health worker cadre due to their higher-level training requirements and scope of work.

## CONCLUSIONS

The review identified 23 CHW cadres that vaccinated in 20 countries. These vaccinating CHW cadres support equity in immunization access for rural and under-reached communities. CHWs who vaccinate face the following challenges: (1) inadequate supply chain training, (2) inadequate cold chain equipment, (3) transportation for supplies and to communities, (4) heavy existing workload, (5) inadequate or irregular remuneration, (6) inadequate or irregular supervision. To improve immunization coverage in underimmunized and zero-dose communities, countries where CHWs vaccinate should provide CHWs with adequate remuneration, supervision, supply chain support and management, and formal integration within the health system.

CHWs administered vaccines in 20 of the 75 countries with documented CHW programs, suggesting the majority of an estimated 3.3 million CHWs globally do not yet administer vaccines.^78^^,^^79^ In light of health care workforce shortages and immunization equity gaps, further exacerbated by the COVID-19 pandemic, there may be settings where task-shifting vaccine administration to CHWs should be considered to bolster immunization access for under-reached communities. To further explore best practices to support CHWs as vaccinators and encourage task-shifting in those countries where CHWs do not currently vaccinate, additional systematic documentation is needed, especially related to supply chain, policy, safety, and efficacy.

## Supplementary Material

GHSP-D-22-00307-Supplements.pdf

## Notes

**Peer Reviewed**

**First published online:** February 14, 2023.
