# Supplementary material for: Community Health Workers as Vaccinators: A Rapid Review of the Global Landscape, 2000–2021
Source: Glob Health Sci Pract. 2023 Feb 28;11(1):e2200307. doi: 10.9745/GHSP-D-22-00307 (PMC9972374; doi:10.9745/GHSP-D-22-00307)
Supplement: GHSP-D-22-00307-Supplements.pdf [file GHSP-D-22-00307-Supplements.pdf]

Supplement to: Gibson E, Zameer M, Alban R, Kouwanou LM. Community health workers as vaccinators: a rapid review of the global landscape (2000-2021). *Glob Health Sci Pract*. 2023;11(1):e2200307.
https://doi.org/10.9745/GHSP-D-22-00307

SUPPLEMENT 1. Database Search Terms for the Rapid Review of Community Health Workers as Vaccinators

**Database,** Results<br><br>Filters	Search Terms
**PubMed,** 175<br><br>Title/Abstract, filter by year (2000-2021)	((aides, community health[MeSH Terms]) OR (community health worker[MeSH Terms]) OR (community health workers[MeSH Terms]) OR (aides, community health[MeSH Terms]) OR (traditional birth attendant[MeSH Terms]) OR (traditional birth attendants[MeSH Terms]) OR (community health worker*[Title/Abstract]) OR (health extension worker*[Title/Abstract]) OR (health development army[Title/Abstract]) OR (agents de santé communautaire[Title/Abstract]) OR (agent de santé communautaire[Title/Abstract]) OR (Health surveillance Agent*[Title/Abstract]) OR (community health volunteer*[Title/Abstract]) OR (Family Welfare Assistant*[Title/Abstract]) OR (Health assistant*[Title/Abstract]) OR (Community health care provider*[Title/Abstract]) OR (community birth attendant*[Title/Abstract]) OR (Lady Health Worker*[Title/Abstract]) OR (Health Surveillance Assistant*[Title/Abstract]) OR (Anganwadi Worker*[Title/Abstract]) OR (Accredited Social Health Activist*[Title/Abstract]) OR (Accompagnateur*[Title/Abstract])OR (Shasthya Shebika*[Title/Abstract]) OR (Health Assistant*[Title/Abstract]) OR (Community-Based Skilled Birth Attendant*[Title/Abstract]) OR (Community Health Agent*[Title/Abstract]) OR (Community Health Assistant*[Title/Abstract]) OR (Community Based Agent*[Title/Abstract]) OR (Village Health Worker*[Title/Abstract]) OR (Village Health Team*[Title/Abstract]) OR (Home Based Carer*[Title/Abstract]) OR (Female Community Health Volunteer*[Title/Abstract]) OR (Maternal Child Health Worker*[Title/Abstract]) OR (Behvarz*[Title/Abstract]) OR (Brigadista*[Title/Abstract]) OR (Volunteer Midwives*[Title/Abstract]) OR (Volunteer Collaboration*[Title/Abstract]) OR (Health Promoter*[Title/Abstract]) OR (Agentes Polivalent* Elementar*[Title/Abstract])) AND ((administ*[Title/Abstract]) OR (deliver*[Title/Abstract]) OR (inject*[Title/Abstract]) OR (give*[Title/Abstract]) OR (gave[Title/Abstract]))
**Cochrane Library (Reviews and Trials),** 3 reviews, 111 trials	("community health worker" OR "community health aid" OR "traditional birth attendant" OR "health extension worker" OR "health surveillance agent" OR "community health volunteer" OR "family welfare assistant" OR "health assistant" OR "community health care

Supplement to: Gibson E, Zameer M, Alban R, Kouwanou LM. Community health workers as vaccinators: a rapid review of the global landscape (2000-2021). *Glob Health Sci Pract*. 2023;11(1):e2200307.
https://doi.org/10.9745/GHSP-D-22-00307

**Database,**<br>Results<br><br>Filters	Search Terms
Title/Abstract/Keyword (default), restricted to 01/01/2000 to 30/07/2021	provider” OR “community birth attendant” OR “lady health worker attendant” OR “health extension worker” OR “health surveillance agent” OR “co” OR “health surveillance assistant” OR “Anganwadi Worker” OR “Accredited Social Health Activist” OR “Accompagnateur” OR “Shasthya Shebika” OR “Health Assistant” OR “Community-Based Skilled Birth Attendant” OR “Community Health Agent” OR “Community Health Assistant” OR “Community Based Agent” OR “Village Health Worker” OR “Village Health Team” OR “Home Based Carer” OR “Female Community Health Volunteer” OR “Maternal Child Health Worker” OR “Behvarz” OR “Brigadista” OR “Volunteer Midwives” OR “Volunteer Collaboration” OR “Health Promoter” OR “Agentes Polivalentes Elementares”):ti,ab,kw AND (vaccination OR immunization):ti,ab,kw AND (administer OR deliver OR inject OR give OR gave):ti,ab,kw" (Word variations have been searched)
**WebOfScience:**<br>143<br><br>Abstract, 2000-2021	((AB=(“community health worker*” OR “community health aid*” OR “traditional birth attendant*” OR “health extension worker*” OR “health surveillance agent*” OR “community health volunteer*” OR “family welfare assistant*” OR “health assistant*” OR “community health care provider*” OR “community birth attendant*” OR “lady health worker*” OR “health surveillance assistant*” OR “Anganwadi Worker*” OR “Accredited Social Health Activist*” OR “Accompagnateur*” OR “Shasthya Shebika*” OR “Health Assistant*” OR “Community-Based Skilled Birth Attendant*” OR “Community Health Agent*” OR “Community Health Assistant*” OR “Community Based Agent*” OR “Village Health Worker*” OR “Village Health Team*” OR “Home Based Carer*” OR “Female Community Health Volunteer*” OR “Maternal Child Health Worker*” OR “Behvarz*” OR “Brigadista*” OR “Volunteer Midwives*” OR “Volunteer Collaboration*” OR “Health Promoter*” OR “Agentes Polivalent* Elementar*”)) AND AB=(vaccin* OR immuniz* OR immunis*)) AND AB=(administer* OR deliver* OR inject* OR give* OR gave)

Supplement to: Gibson E, Zameer M, Alban R, Kouwanou LM. Community health workers as vaccinators: a rapid review of the global landscape (2000-2021). *Glob Health Sci Pract*. 2023;11(1):e2200307. https://doi.org/10.9745/GHSP-D-22-00307

SUPPLEMENT 2. Selected Documents for the Rapid Review of CHWs as Vaccinators

**Author(s)**<br>**Year**	**Article Type**	**Vaccinating CHW Cadres, Countries Identified**
Admassie, Abebaw, and Woldemichael[38]<br>2009	Original research, impact evaluation	Health Extension Worker (HEW), Ethiopia
Ahmed et al.[39]<br>2021	Report	Community Health Volunteer (CHV), Kenya
Bilal et al.[40]<br>2011	Report	Health Extension Worker (HEW), Ethiopia
Bisrat et al.[41]<br>2019	Original research, cross sectional survey	Health Extension Worker (HEW), Ethiopia
Chernoff and Cueva[42]<br>2017	Original research, qualitative methods	Community Health Aids/Practitioners (CHA/Ps), United States
Closser[43]<br>2015	Opinion	Lady Health Worker (LHW), Pakistan
Gavi: The Vaccine Alliance[44]<br>2018	Report	Lady Health Worker (LHW), Pakistan
Glenton et al.[45]<br>2013	Review	Village Health Volunteer, Papua New Guinea<br>Traditional Birth Attendant (TBA), Ghana<br>Traditional Birth Attendant (TBA), Mali
Gov. of the Republic of Malawi: Ministry of Health[23]<br>2017	Report	Health Surveillance Assistant (HSA), Malawi
Hobble, Rosqueta, and McLaughlin[46]<br>2018	Report	Community Health Worker (CHW), Kenya
Hodgins[47]<br>2021	Report	Health Assistant (HA), Bangladesh<br>Auxiliary Health Worker (AHW), Nepal<br>Health Extension Worker (HEW), Ethiopia<br>Community Health Extension Workers (CHEWs) or Junior CHEWs, Nigeria
Huang et al.[48]<br>2018	Review	Village-based Health Worker, China

Supplement to: Gibson E, Zameer M, Alban R, Kouwanou LM. Community health workers as vaccinators: a rapid review of the global landscape (2000-2021). *Glob Health Sci Pract*. 2023;11(1):e2200307.
https://doi.org/10.9745/GHSP-D-22-00307

**Author(s)**<br>**Year**	**Article Type**	**Vaccinating CHW Cadres, Countries Identified**
Kabambi and Abdallah[49]<br>2020	Editorial	Volunteer Vaccinators, Democratic Republic of Congo
Krishnaratne et al.[50]<br>2021	Original research, evaluation	Health Extension Worker (HEW), Ethiopia
Levin et al.[51]<br>2005	Original research, retrospective cost analysis	Village-based Midwife, Indonesia<br>Traditional Birth Attendant, Afghanistan<br>Traditional Birth Attendant, Mali
Mavalankar and Vora[52]<br>2008	Report	Auxiliary Nurse Midwife (ANM), India
Munthali[53]<br>2016	Report	Health Surveillance Assistant (HSA), Malawi
Murakami et al.[54]<br>2014	Original research, mixed methods	Lady Health Worker (LHW), Pakistan
Nath, Kaur, and Tripathi[55]<br>2015	Original research, site evaluation and cross-sectional survey	Auxiliary Nurse Midwife (ANM), India
Ngwira, Hutchinson, and Mayhew[56]<br>2021	Original research, qualitative methods	Health Surveillance Assistant (HSA), Malawi
Nicks et al.[57]<br>2021	Opinion	Health Surveillance Assistant (HSA), Malawi
Nyirenda and Flikke[58]<br>2013	Original research, qualitative methods	Health Surveillance Assistant (HSA), Malawi
Perry[59]<br>2021	Report	Health Extension Worker (HEW), Ethiopia<br>Auxiliary Nurse Midwife (ANM), India<br>Health Assistant (HA), Bangladesh<br>Community Health Extension Worker (CHEW), Nigeria<br>Lady Health Worker (LHW), Pakistan<br>Health Surveillance Assistant (HSA), Malawi<br>Community Health Agent (CHA), Brazil<br>Behvarzs, Iran<br>Community Health Nurse (CHN) and Community

Supplement to: Gibson E, Zameer M, Alban R, Kouwanou LM. Community health workers as vaccinators: a rapid review of the global landscape (2000-2021). *Glob Health Sci Pract*. 2023;11(1):e2200307.
https://doi.org/10.9745/GHSP-D-22-00307

**Author(s) Year**	**Article Type**	**Vaccinating CHW Cadres, Countries Identified**
		Health Officer (CHO), Ghana<br>Kader, Indonesia<br>Agents de Santé Communautaire (ASC), Niger
Prinja et al.[60] 2014	Original research, economic analysis	Auxiliary Nurse Midwife (ANM), India
Punjab University Institute of Communication Studies[21] 2011	Evaluation	Lady Health Worker (LHW), Pakistan
Sebhatu[61] 2008	Report	Health Extension Worker (HEW), Ethiopia
Singh et al.[62] 2018	Original research, mixed methods time and motion study	Auxiliary Nurse Midwife (ANM), India
Singh et al.[63] 2015	Original research, case studies	Behvarz, Iran
Thacker et al.[64] 2013	Original research, cross sectional survey	Auxiliary Nurse Midwife (ANM), India
Tsega et al.[65] 2016	Original research, cross sectional descriptive cluster survey	Health Surveillance Assistant (HSA), Malawi
USAID Maternal and Child Survival Program[66] 2018	Report	Auxiliary Nurse Midwife (ANM), India
Wang et al.[67] 2007	Program evaluation	Village-Based Health Worker, China
